# Advancing Equity in Living Donor Kidney Transplant: We Need More Male Donors

**DOI:** 10.34067/KID.0000000632

**Published:** 2024-12-26

**Authors:** Jessica L. Harding

**Affiliations:** Department of Surgery, Emory University School of Medicine, Atlanta, Georgia; Department of Epidemiology, Rollins School of Public Health, Emory University, Atlanta, Georgia; and Health Services Research Center, Emory University School of Medicine, Atlanta, Georgia

**Keywords:** gender difference, kidney transplantation

Globally, women are more likely to donate than receive a living kidney donor transplant (LDKT). Specifically, women constitute approximately 60% of LDKT donors, yet <40% of LDKT recipients.^1^ Although there is no conclusive evidence why women donate more than they receive, this inequality likely stems from complex interactions between medical, socioeconomic, cultural, and cognitive or emotional factors.^1^

With just a few exceptions,^2,3^ most studies examining sex imbalances in LDKT do so among individuals who have already undergone donor nephrectomy because these data are often readily available from national surveillance systems and donor registries. However, the exclusion of potential living donors (PLDs) who may have been willing to donate but did not, for various reasons, make it through the evaluation process limits our ability to draw causal inference on the reasons for sex inequities in LDKT. Examining sex inequities in LDKT among PLDs (including donors and nondonors) would allow us to answer the question: Are men and women similarly likely to want to donate, but differentially excluded during the evaluation process because of, for example, medical contraindications as is hypothesized?

In this month's issue of *Kidney360*, Chumdermpadetsuk *et al.*^4^ examine sex differences in the likelihood of men and women making it through key LDKT evaluation steps among PLDs self-referring to a single transplant center in Massachusetts, United States. Between 2009 and 2022, they identified 1861 adult PLDs from the Organ Transplant Tracking Record database who had completed the self-referral evaluation form. There were eight key binary outcomes (yes/no) of reasons for being excluded as a PLD captured from extensive chart review: (*1*) undergoing blood testing; (*2*) being ABO blood type incompatible with the recipient; (*3*) having a positive cross-match with the recipient; (*4*) completing medical and/or psychosocial workup; (*5*) having a medical and/or psychosocial contraindication; (*6*) recipient too sick, deceased, or declined gift; (*7*) being approved for donation; and (*8*) proceeding with the donation.^4^ At this center, PLDs were deemed ineligible if they had diabetes or HIV, were taking >2 medications for hypertension, had body mass index >32 kg/m^2^, had more than one lifetime occurrence of nephrolithiasis, or had concern for malignancy.

Overall, Chumdermpadetsuk *et al.*^4^ found that women were almost twice as likely to complete a self-referral form for LDKT evaluation than men (65.2% versus 34.8% of all PLDs), but similarly likely to donate (6.3% versus 7.6%). Among self-referred PLDs, men and women were equally likely to undergo blood testing, have a positive cross-match with the recipient, have complete medical and/or psychosocial workup, have a medical and/or psychosocial contraindication (although these were different by sex), and be approved for and proceed with donation, after adjustment for donor age, race, ethnicity, relationship with the recipient, and recipient sex. There were only two notable differences. First, female PLDs were approximately twice as likely (adjusted relative risk, 1.93 [1.34–2.77]) to have ABO incompatibility with their intended recipients, and second, female PLDs were 2.5 times as likely (2.51 [1.18–5.36]) to be excluded because of the recipient being too sick, deceased, or declined gift.^4^ Importantly, these last two factors would favor male donation and do not explain female overrepresentation among PLDs.

Collectively, these findings largely support the premise that men are not more likely to be excluded as PLD because of medical contraindications or lack of progression through the evaluation process for other reasons as was previously thought, but rather they are not volunteering to be PLDs to the same extent as women. One important caveat to this interpretation is that the study population included only those who completed the self-referral intake form. It is understood that once a PLD clicks the online intake form (*i.e*., starts the referral process), they are informed that they are not eligible for organ donation if they have diabetes, HIV, or hypertension. It is possible that male PLDs may have been more likely to have one of these comorbidities and thus not completed the intake form and, therefore, be underrepresented among the PLDs captured in the current study. Certainly, it is true in the general population that men are more likely to have diabetes, hypertension, and HIV than women. Unfortunately, data on the demographics of those who did not complete the self-referral intake form are missing. Being able to track individuals as they progress through the donation process is challenging, but key to answering questions of who is most likely to consider becoming a PLD. It is unclear from the current study whether this statement on eligibility is shown to PLDs before or after any demographic data are captured during the intake referral process. If the latter, there may be opportunity to examine the sex distribution among all PLDs accessing the form. If the former, there is likely opportunity to redesign this form to facilitate better tracking of all PLDs across the continuum of the referral process.

Nonetheless, this study is the most comprehensive quantitative study to date to indicate that men may be less likely to consider being a PLD than women. Reasons for this are complex, but a 2023 review of sex inequities in LDKT sheds some light.^1^ In this review, four studies from North America showed that economic factors, namely the primary income-earning role of men in families, may be key drivers of why men are less likely to consider donation.^1^ A population-based study from the United States found that the overall donation rate declined in men, but not in women, between 2005 and 2015, ^5^ with income effects on donor rates much more pronounced in men compared with women.^5^ A qualitative study from Israel found that mothers were the dominant donors, in part to protect their husbands who were the sole income earners for the family.^6^ Insurance is also likely to play a role because men are less likely to have insurance, which is likely to pose a significant barrier to donation.^7^ In the study by Chumdermpadetsuk *et al.*, there was no significant difference in the education level, employment status, and expression of financial concern between male and female donors, although in line with other studies,^7^ male donors were significantly less likely to have health insurance. Unfortunately, even in this study, findings on socioeconomic status were captured only among those who progressed to donation (where more information is documented) and were not captured among non-donors who were excluded during the evaluation process. Again, designing referral intake forms that prioritize the capture of this information as early in the referral process as possible will be key to disentangling some of these important questions related to the demographic profile of all PLDs.

For women, the decision to donate a kidney is likely driven by a desire to improve the life and health of recipients, stemming from the traditional caregiving roles that women have typically occupied in society.^8^ Research shows that a greater proportion of women are willing to offer their kidneys to both families and strangers as compared with men and are, in general, less willing to accept any financial compensation for LDKT, including health insurance coverage.^1^ Globally, mothers donate more often than fathers.^1^ In the study by Chumdermpadetsuk *et al.*, female (versus male) PLDs were more likely to have “non-related other” as their relationship status to the recipient, a group consisting of nonspousal, nonbiologically related individuals, such as friends, in-laws, and community members, suggestive of a wider network of relational duty and commitment.^4^

The key contribution of the study by Chumdermpadetsuk *et al.* is the examination of all PLDs regardless of whether this resulted in donation. Their findings suggest that there may be an “untapped pool of male individuals who are not currently being considered for donation, but would likely be eligible.” Future research is needed to identify the causes of this phenomenon to inform the design of targeted interventions for increasing male living donation, thus expanding access to LDKT for all. In the meantime, expanding financial assistance to alleviate the unforeseeable financial burden and out-of-pocket costs associated with the donation process will likely be key.^9^ This could include legislative protection from job loss and insurance discrimination, tax credit on actual donor costs, reimbursing lost wages, and childcare and eldercare expenses.^9^ Universal health insurance, a lofty goal in the United States, may also increase the pool of men who would consider being a PLD. Finally, encouraging men to play more of an integral caring role within families and societies at large may in turn increase their motivation for donation. This is also likely to be alleviated with upstream system-level policies, such as paternity leave, that will advance equity in the way we care for each other in society, including through organ donation.
